# Scotopic Microperimetry in the Early Diagnosis of Age-Related Macular Degeneration: Preliminary Study

**DOI:** 10.1155/2014/671529

**Published:** 2014-12-09

**Authors:** Marcella Nebbioso, Andrea Barbato, Nicola Pescosolido

**Affiliations:** ^1^Department of Sense Organs, Faculty of Medicine and Odontology, Policlinico Umberto I, Sapienza University of Rome, Piazzale Aldo Moro 5, 00185 Rome, Italy; ^2^Department of Sense Organs, Ocular Electrophysiology Center, Sapienza University of Rome, Viale del Policlinico 155, 00161 Rome, Italy; ^3^Department of Cardiovascular, Respiratory, Nephrology, Geriatric, and Anesthetic Sciences, Faculty of Medicine and Odontology, Policlinico Umberto I, Sapienza University of Rome, Piazzale Aldo Moro 5, 00185 Rome, Italy

## Abstract

*Background*. Recent clinical studies have shown that, in some degenerative retinal diseases, like age-related macular degeneration (AMD), the sensitivity of the rods decreases more rapidly than the sensitivity of the cones. The aim of this study was to evaluate if there is a correlation between the presence of hard drusen at the macular level and the rod damage responsible for the reduction in scotopic retinal sensitivity in subjects at risk for AMD. *Methods*. The authors selected 24 subjects (14 men and 10 women) with an average age of 67.25 ± 5.7 years. Macular hard drusen were present in 50% of the subjects at the *fundus oculi* exam. The researchers evaluated the retinal sensitivity to light in mesopic and scotopic conditions of each subject with an MP-1 scotopic microperimeter (MP-1S). *Results*. In subjects with hard drusen in the *fundus oculi* examination, there was a statistically significant reduction in scotopic retinal sensitivity, while the mesopic retinal sensitivity was not compromised. *Conclusion*. This study revealed how the presence of hard drusen at the macular level is associated with a reduction in scotopic retinal sensitivity compared to a control group of healthy subjects. Retinal functionality in a scotopic setting examined with MP-1S could be useful in early diagnosis of AMD.

## 1. Introduction 

Recent clinical studies have shown that in some degenerative retinal diseases the sensitivity of the rods decreases more rapidly than the sensitivity of the cones. Also chronically high stress, cellular hypoxia, and impaired autophagy (or self-eating) can induce retinal pathological changes which may be irreversible, like age-related macular degeneration [1–3]. Oxidative damage of the cellular components, primary source of reactive oxygen species, plays an important role in the senescence process and impaired lysosomal function evokes decreased autophagy flux that may be a critical aspect of retinal pigment epithelium (RPE) cell degeneration and age-related macular degeneration development [2].

In clinical studies, rod function is measured by scotopic electroretinogram, or by dark-adapted perimetry, for which the most used instrument is the Humphrey Field Analyzer (HFA) (Carl Zeiss Meditec Inc., USA) by means of “5.25 floppy discs and a light-pen.” However, this system and the second generation HFA are not capable of correcting errors related to poor fixation by the patient. Scotopic microperimetry is a promising new diagnostic method which can evaluate rod function and is an evolution of standard MP-1 microperimetry (Nidek Technologies, Italy, 2002). The MP-1 is essentially an instrument which has recently made possible the integration of the subjective data of computerized microperimetry and the objective data of retinal images, thus allowing precise measurements of the macular function within the central 45 degrees [2–4].

In healthy subjects, in dim light, there is a physiological central scotoma, corresponding to the foveal area in which there are no rods. This retinal region measures approximately 0.35 mm and corresponds approximately to the central 1.25°. A high density of rods is instead found around the central 5°-6° such that following dark adaptation greater sensitivity is observed in this retinal area [1, 3].

Rod sensitivity is compromised in many retinal pathologies like pigmentary retinopathy, rod-cone dystrophy, retinal telangiectasia, congenital night blindness, and central serous chorioretinitis. Recent histological evidence, moreover, shows that rods can be damaged earlier than cones in the early stages of advanced macular degeneration (AMD), particularly at the parafoveal level where they are found in greater numbers [5–7]. In line with this morphological data, there is evidence that affected subjects prematurely present difficulty in adapting to twilight vision [5–10].

AMD, the main cause of irreversible central vision function deficiency in developed countries among people over 50 [4, 6], includes geographic atrophy, choroidal neovascularization, and detachment of RPE [11–13]. This is the most advanced stage of age-related maculopathy (ARM), a frequent condition in the elderly characterized by the presence of macular soft drusen (with dimensions over 63 *μ*m) and/or areas of macular hyper/hypopigmentation of the EPR. Clinical studies have demonstrated that, although in many cases the presence of drusen is associated with normal visual function, a significant number of subjects with macular drusen present a reduction in central vision, especially in dim light [4, 14–17]. The exact role of drusen in the pathogenesis of AMD is still not entirely clear. Currently, soft and/or confluent drusen are a definite sign of AMD and their presence implies a great increase in the risk of reduced visual acuity, especially in conditions of dim light, while hard drusen do not seem to have significant clinical relevance in the pathogenesis of this degenerative disease [5, 6, 18].

The aim of the research was to study the retinal sensitivity in mesopic and scotopic conditions with MP-1 in patients presenting hard drusen at the* fundus oculi* exam* versus* normal subjects in order to evaluate how premature the reduction of the scotopic function is compared to the mesopic one. In fact, this clinical condition is usually considered a mere accentuation of the normal aging process but also the first pathological aspect in patients that could be at risk of AMD.

## 2. Methods

We selected 24 eyes of 24 subjects (14 males and 10 females) with an average age of 67.25 (±5.7) years for the study. In accordance with the declaration of Helsinki, we informed each subject regarding all aspects of collected data use and they signed the written consent prior to enrollment. The Ethics Committee of Sapienza University of Rome approved the protocol of the study.

All the individuals were asymptomatic for ocular pathology and had normal medical histories, with no sign of systemic hypertension, diabetes, or other concomitant pharmacological treatment. None of the subjects showed refractive disturbances more than ±4.00 D sphere or concomitant ocular diseases such as retinal scars, cataracts, retinal vasculitis, or glaucoma. All the 12 controls (8 men and 4 women) had normal ocular examinations and negative* fundus oculi,* while macular hard drusen, with dimensions under 63 *μ*m, were present in 12 of the sample at the ophthalmoscopic exam. The ocular assessments were all within normal range: visual acuity (20/20), colour perception (Ishihara chart), Amsler grid, intraocular pressure, slit lamp, fundoscopy, and visual field HFA 30-2 program. Both the investigators and the individuals studied were blinded to the diagnosis.

All subjects underwent pupil dilation through administration of tropicamide 1% drops. After 20 minutes we performed a microperimetry test with MP-1 in mesopic conditions. Then, the subjects waited in a dark room for 30 minutes prior to performing MP-1 in scotopic conditions.

*MP-1 scotopic microperimetry (MP-1S)* is the modified version of the MP-1 (Nidek Technologies, Italy, 2002), with all the original functions plus the possibility of measuring rod sensitivity following dark adaptation. It thus allows for simple evaluation of the threshold of retinal sensitivity to light both in mesopic and scotopic conditions. To evaluate the scotopic retinal sensitivity, the MP-1S must reduce the luminance of the entire system.

So, standard mesopic microperimetry (MP-1) uses a background luminance of 1.27 cd/m^2^ (equal to 4 asb) with retinal light sensitivity of the range of the stimuli from 0 to 20 dB (0–2 UL). Then, in order to guarantee the dim light conditions necessary for stimulating rod function, all light emitted from the instrument and the computer is blocked out by a light shield made of black fabric between the optical head and the chin rest to protect the patient from accidental exposure to external light. The MP-1S is also equipped for scotopic use with a couple of additional filters that extend the dynamic array of the instrument luminosity to the very subdued illumination conditions required: the first one consists of a 2.0 log unit neutral filter that annuls the 4 asb of luminance of MP-1 mesopic and, therefore, reduces screen luminance. The second one is a 500 nm short pass filter, rod specific sensitivity wavelength, which prevents the stimulation of the cones of the dark-adapted patient. The pair of filters is contained inside a sliding support, or “slider,” which can be manually inserted or removed by the operator. The exam must be conducted in a dark room with a light level of less than 0.1 lux. In detail the instrument characteristics are the following:

1. retinal eye-tracking based on real-time analysis of morphological structures and of the patient's fixation during the exam;
2. automatic static/dynamic projection of stimuli definable in form, color, and size with customization of tests in terms of number, type, and intensity of the stimuli;
3. ability to follow-up with accurate and reproducible retests of the previously acquired information;
4. visualization of differential maps to evaluate sensitivity variations and high resolution and precision scotometry;
5. acquisition of color retinographies in nonmydriatic mode and use of retinal images coming from other technologies to overlap functional data;
6. visual rehabilitation mode through auditory feedback and visual stimulation correlated to training of fixation for improvement of vision quality;
7. scotopic mode to analyze the selective functional response of rods without the response of cones.

The microperimetric map was created using a specific predefined pattern made up of 39 regularly spaced points inside a central 6° circle excluding 1° from the center of fixation in which the fixation target was located. The fixation target was represented by a single cross projected on the central 2° for mesopic microperimetry or a circle included in the central 2° for scotopic microperimetry. The stimulus was a Goldmann size III target presented for 200 msec (Table 1). We determined the sensitivity of each point using a 4-2 strategy. Once we completed the exam, the lighting in the room was returned to normal and we took a color photograph of the* fundus* in order to record the microperimetry data on it.

### 2.1. Statistical Evaluation

We expressed all the variables as mean ± SD when appropriate, taking into account the average of the retinal sensitivity values at the various points of analysis. In this preliminary research we studied 24 subjects and used unpaired Student's *t*-test for 2 independent samples to compare parametric variables between groups. The statistical significance of the comparison was attested to a probability value of *P* < 0.05. 

## 3. Results

Mean age of the patients was 66.37 ± 3.4 years and of the controls was 67.1 ± 7.5 years with a range of 63 to 72 years (Table 2). In order to minimize the learning effect, we recalled the subjects for a follow-up assessment the next week. The two groups were resubmitted to MP-1 and MP-1S exams and we used only the last data for statistical analysis. We calculated the mean values for the two retinal sensitivity tests to light in both mesopic and scotopic conditions.

**Figure 1:**
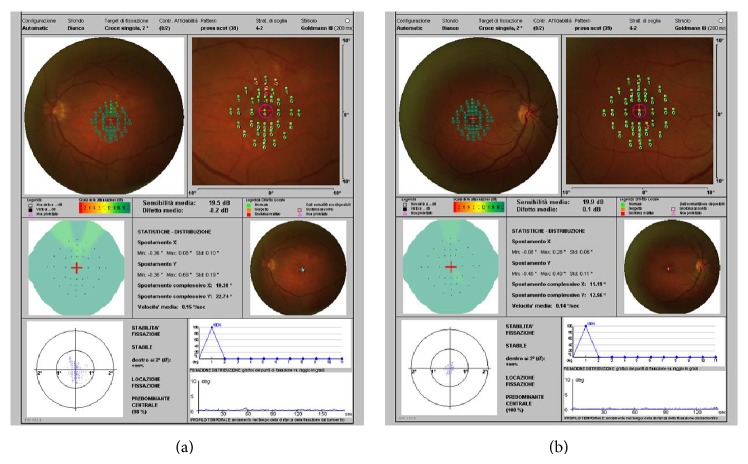
Mesopic microperimetry (MP-1). (a) Patient with hard drusen. (b) Control. The results of the two groups were not statistically significant.

We evaluated retinal sensitivity through a mesopic microperimetry map created using a pattern of 39 points. We found sensitivity was not compromised in both groups. In fact, the average value was 19.07 ± 0.21 dB in the patients with hard drusen and 19.17 ± 0.99 dB in the controls without* fundus* alterations (Figure 1). Therefore, the results of the two groups were not statistically significant (Table 2).

**Figure 2:**
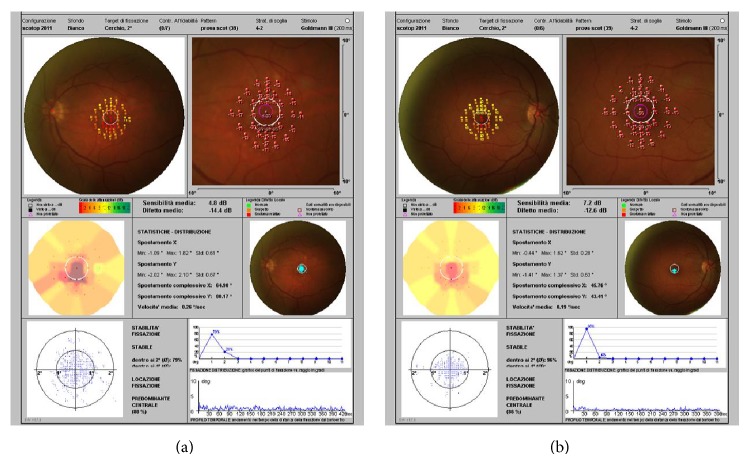
Scotopic microperimetry (MP-1S). (a) Patient with hard drusen. (b) Control. The results were statistically significant (*P* < 0.003).

Rather, we found a marked reduction in scotopic retinal sensitivity in the patients with hard drusen compared with the control group (Figure 2), 5.20 ± 1.18 dB versus 7.70 ± 0.24 dB, respectively. This reduction was found to be statistically significant (*P* < 0.003) (Table 2).

## 4. Discussion

This study was aimed to evaluate the possible correlation between the presence of hard drusen at the macular level and reduction of scotopic sensitivity, in order to obtain early indications of the risk to develop AMD through scotopic microperimetry MP-1S.

We know that, in the adult, from the age of 34, there is a constant decline in the number of rods, approximately 2 rods/mm^2^ every day [19, 20]. More recent histological studies have shown that with advancing age the external segments of the cones and rods tend to become disorganized and refracting bodies, probably made up of lipofuscin [4–6], accumulated inside the internal segments of the cones. An irregular loss of photoreceptor nuclei has also been found, which seems inversely correlated with the age of the subjects and with the lipofuscin levels on the adjacent RPE [21–26].

However, in subjects without macroscopically evident retinal alterations, said phenomenon does not alter the photoreceptor layer structure. This structure appears to be qualitatively normal, as the internal segments of the “surviving” rods expand, not allowing the formation of gaps or insertion of other cells [27]. Thus, a healthy retina of an elderly subject appears histologically similar to that of a young subject.

Scholl et al. observed that in subjects with ARM there were significant retinal areas with an increase in* fundus* autofluorescence (FAF). In these regions there was a consistent reduction in scotopic retinal sensitivity measured with the HFA, while the mesopic retinal sensitivity resulted normal or slightly reduced. This suggests that the increase of FAF in the course of ARM is correlated with a preferential dysfunction of the rod system [28]. The FAF would depend mainly on the accumulation of lipofuscin inside the RPE, as demonstrated by Delori et al. [29].

With aging and in the course of ARM a prevalent deterioration of the rod system occurs. The mechanism of said deterioration could be linked even to the selective vulnerability of the rods themselves. Indeed, the turnover of its outer segments containing photosensitive chemicals requires a high expenditure of energy both for the disposal of “old” disks with rhodopsin by the RPE and for their regeneration. Every day nearly 10–15% of the disks of each rod is phagocytized and replaced by the RPE cells, and a high turnover particularly involves the parafoveal rods [4–6, 14, 15]. Hence, the RPE is responsible for the integrity of the photoreceptor system. The changes of the age-related photoreceptors, or a dysfunction of the RPE, or an accumulation of lipofuscin granules at its level would thus cause a slowdown in photopigment formation and an increase in the circumvolutions of the rod disks with a resulting suffering and loss of cells, especially in those retinal areas with greater density, or between 3 and 5 mm from the center of the fovea [5, 6].

Curcio et al. have come to different conclusions since the loss of rods would be found between 0.5 and 3 mm, thus in an area closer to the fovea. In particular, there was a lack of correspondence between the distribution of the hard drusen and the FAF. The dysfunction of the photoreceptors would thus follow the distribution of the FAF and not that of the drusen, suggesting that there are two different expressions of ARM [14, 15]. The confluent soft drusen correspond to small areas where the RPE is detached and thus the scotopic sensitivity is reduced, as demonstrated even by Owsley et al. [7].

Still, Jackson et al. have demonstrated that subjects with initial ARM do not respond abnormally in the “a” wave of the scotopic electroretinogram (ERG) [20, 30]. To understand these results it must be remembered that in ARM the dysfunction or loss of rods in a very restricted area like the macula seems to have a minimal impact on the massive response of the “a” wave of the rods [30]. However, we cannot exclude the possibility that the activation of the response of the rods in the central retina is abnormal and that the measuring of the parameters of the “a” wave of the rods-mediated ERG would reveal abnormalities [14, 15]. A result in that sense could be obtained through multifocal electroretinography (mfERG).

Feigl et al. did indeed demonstrate a significant delay in the average response of rods in mfERG in patients with early ARM compared to the control group, while the average cone-mediated responses were within the normal range [31]. According to the authors, the rod-mediated mfERG with dark-adaptation would be the most sensitive test in recording functional retina deficits in ARM. The limit of this exam seems however to be the fact that the mfERG fully reflects the bipolar rod cell activity, not that of the photoreceptor itself [32, 33]. It has yet to be determined if this technique can be reliably and validly implemented in the studies on ARM pathogenesis [30, 31, 34].

As far as our results are concerned, the group of subjects presenting small hard drusen on the fovea showed, despite good visual acuity, an altered dark-adaptation compared to the healthy, same-aged control group. In fact, the MP-1S test showed a reduction in retinal sensitivity measured in scotopic conditions in the patients group.

The role of hard drusen in the etiopathogenesis of AMD is still debated as a physiological expression of aging, but it could instead be an important predictive factor for the development of this pathology.

Our study leads us to hypothesize that the damage to the RPE verified in the course of ARM is caused, firstly, by the suffering of the rods which translates into compromised night vision. It is only at a later point that the cones become involved, following damage to the rods. To explain this variation in timing of photoreceptor damage, it must be remembered that rods and cones have different visual cycles. While in the outer segment of each photoreceptor the reduction of the all-*trans* retinal to all-*trans* retinol occurs, all the other reactions including isomerization occur in the RPE cells for the rods and in the Müller cells for the cones [30].

Many protein and lipid constituents of drusen are similar to those found in deposits characteristic of specific forms of amyloid that are known to be pathogenic in a variety of neurodegenerative diseases and other age-related degenerative disorders such as Alzheimer disease and other amyloid diseases (fatty liver, skin thinning, atherosclerosis, elastosis, amyloidosis, and osteoporosis) [35]. Deposition of these structures may lead to local toxicity of RPE or induction of local inflammatory events that contribute to drusen biogenesis and the pathogenesis of AMD [35]. Despite intensive studies on amyloid assembly* in vitro* and* in vivo*, the localization and cellular mechanisms of amyloid oligomerization are not fully understood [36].

Although small hard drusen are currently considered innocuous and of little clinical relevance to the genesis of AMD, our results would confirm the hypothesis according to which the presence of such macular lesions could be a factor to be taken into consideration in the development of AMD in subjects whose mesopic function is not yet compromised. This strengthens the hypothesis that there is a correlation between hard drusen and rod dysfunction.

Our results, as far as normal subjects are concerned, are in line with those of Crossland et al. [1]. Nonetheless, the introduction of scotopic microperimetry in clinical practice is suggested to prevent further functional and organic retinal damage as much as possible.

In summary, we will continue the research on elderly subjects to verify our hypothesis of the study considering ophthalmological alterations in several AMD and ARM groups of patients with different macular function conditions, even in order to identify the first pathological aspect in the subjects that could be at risk of AMD.

## Figures and Tables

**Table 1:** Technical information of photopic and scotopic microperimetry (MP-1 and MP-1S).

Projection system	Internal LCD monitor.
Background luminance	Mesopic: 1.27 cd/m^2^ (4 asb). Scotopic: 0.0032 cd/m^2^ (0.01 asb).
Stimuli dimensions Stimuli patterns Stimulus duration	Goldmann I, II, III, IV, and V. Preset, manual, and customizable pattern sets. From 40 to 2000 ms (user customizable).
Threshold strategies	4-2-1, 4-2, fast, raw, manual.
Fixation target	Single cross. Circle with “Fade-out Protection” option for MP-1S. Four crosses. Possibility of custom fixation target. Dimensions and colors user customizable in all targets.
Camera	Field of view: 45 degrees. Working distance: 47.1 mm from front lens to patient's eye. Working distance mode: using Purkinje reflex. Minimum pupil diameter: 4 mm. 1/2′′ Infrared CCD camera, 768 × 576 pixel resolution. 1/2′′ Color CCD camera, 1392 × 1040 pixel resolution.

**Table 2:** Data subjects and retinal sensitivity evaluated through mesopic (MP-1) and scotopic microperimetry (MP-1S) map created using a pattern of 39 points. The retinal response, evaluated through mesopic microperimetry, was compromised in both groups. In fact, the average value was 19.07 ± 0.21 dB in the group with hard drusen and 19.17 ± 0.99 dB in the group without *fundus* alterations (not statistically significant). Instead, a marked reduction in scotopic retinal response was found in patients with hard drusen 5.20 ± 1.18 dB versus control group 7.70 ± 0.24 dB (statistically significant, *P* < 0.003).

Subjects	Values	(±)	Values max.	Values min.
Mean age patients	66.37 years	3.4 years	70	63
Mean age controls	67.1 years	7.5 years	72	61
MP-1 patients	19.07 dB	0.21 dB	1.86 dB	0.97 dB
MP-1 controls	19.17 dB	0.99 dB	1.25 dB	0.87 dB
MP-1S patients	5.2 dB	1.18 dB	0.5 dB	0.23 dB
MP-1S controls	7.7 dB	0.24 dB	0.89 dB	0.34 dB
